# The “crossover effect” of COVID-19 in pregnancy on the infant microbiome

**DOI:** 10.3389/fmicb.2025.1569279

**Published:** 2025-05-14

**Authors:** O. Ignatyeva, V. Daniel, E. Zelenova, A. Cherdakli, E. Bolashova, L. Matkava, A. Shegurova, M. Volkov, A. Zagainova, D. Kashtanova, M. Ivanov, B. Bembeeva, V. Zubkov, A. Gordeev, T. Priputnevich, V. Yudin, V. Makarov, A. Keskinov, S. Kraevoy, S. Yudin, V. Skvortsova

**Affiliations:** ^1^Federal State Budgetary Institution “Centre for Strategic Planning and Management of Biomedical Health Risks” of the Federal Biomedical Agency, Moscow, Russia; ^2^Federal State Budgetary Institution “Kulakov National Medical Research Centre for Obstetrics, Gynecology, and Perinatology”, Ministry of Health of the Russian Federation, Moscow, Russia; ^3^Federal Biomedical Agency, Moscow, Russia

**Keywords:** COVID-19, SARS-CoV-2, pregnancy, microbiome, gut

## Abstract

**Background:**

The COVID-19 pandemic has had a significant impact on public health. However, the impact of COVID-19 infection during pregnancy on the microbiome of the mother and her newborn child still remains poorly understood.

**Methods:**

This study involved 94 mother-child pairs whose mothers had COVID-19 during pregnancy and 44 newborns as a control group recruited in 2018. Stool samples were collected from women before delivery and from infants at 5–7 days after birth and used for 16S rRNA sequencing.

**Results:**

We found that the microbiomes of infants exposed in utero to COVID-19 showed decreased microbial diversity and richness. Moreover, we observed a higher inter-sample variability between infant samples in the case group, which might suggest destabilization of their microbiomes. Neither alpha- nor beta-diversity metrics differed significantly between the groups depending on the trimester when the mother contracted COVID-19. Thus, the timing of prenatal COVID-19 exposure had no effect on the infant gut microbiome.

**Conclusion:**

COVID-19 during pregnancy can significantly compromise the establishment of the infant gut microbiome presumably by disrupting the mother’s microbiome.

## 1 Introduction

Although COVID-19 is no longer a public health emergency, it continues to be an ongoing health issue, particularly for certain demographics. Research has shown that pregnant women are highly susceptible to SARS-CoV-2 infection, which may increase the likelihood of pre- and postnatal complications, such as preeclampsia, eclampsia, severe infections, preterm birth, stillbirth, and perinatal mortality (Veerus et al., 2024). Moreover, SARS-CoV-2 infection in expectant mothers has been linked to lower Apgar scores for newborns and an increased risk of neonatal complications (Lv et al., 2024).

The negative effects of COVID-19 are not limited to prenatal and postnatal complications, particularly in cases of moderate and severe infections. It is widely recognized that COVID-19 is linked to dysbiosis in various organ systems, such as the gastrointestinal tract (Zuo et al., 2020; Nandi et al., 2023), respiratory system (Nandi et al., 2023; Ignatyeva et al., 2024; Xie et al., 2024), and urogenital system (Celik et al., 2023). Many studies have shown that SARS-CoV-2 infection can significantly affect commensal bacteria, particularly in the gut, resulting in a considerably lower microbial richness and diversity (Zhang et al., 2023), as well as the depletion of many beneficial species, such as *Bifidobacterium pseudocatenulatum*, *Faecalibacterium prausnitzii* (Liu et al., 2022), and *Akkermansia muciniphila* (Upadhyay et al., 2023). The infection has also been linked to the overgrowth of opportunistic bacteria and fungi, such as the genera *Enterococcus* and *Streptococcus* (Rizzello et al., 2024), *Candida albicans*, and *Aspergillus niger* (Zuo et al., 2020). Thus, SARS-CoV-2 can disrupt the microbiome and lead to various health issues. Moreover, these alterations might be long-lasting, even in mild and asymptomatic cases (Upadhyay et al., 2023; Zhang et al., 2023).

The above effects may have particularly dangerous implications for newborns, as their initial microbiomes are inherited from their mothers (Browne et al., 2022). Any adverse alterations in the mother’s commensal microflora, regardless of the cause, could impact the normal microbial colonization of the newborn’s gut, ultimately affecting its overall health (Yao et al., 2021). A robust microbiome during early life serves as a protective barrier and plays a crucial role in long-term health. However, if disrupted, it becomes susceptible to negative changes that could potentially result in various pathological conditions, such as metabolic syndrome or atopic diseases (Kapourchali and Cresci, 2020).

Despite the dangerous implications of COVID-19 for newborns, there is limited research on its impact on the maternal and neonatal microbiome. The available evidence suggests that SARS-CoV-2 infection during pregnancy results in significant microbial changes, including the nasopharyngeal (Crovetto et al., 2022), oral (Leftwich et al., 2023), gut (Juarez-Castelan et al., 2022; Leftwich et al., 2023), vaginal (Celik et al., 2023; Leftwich et al., 2023), and colostrum microbiomes (Juarez-Castelan et al., 2022). However, findings regarding bacterial diversity and richness are often conflicting. Few available studies have suggested a correlation between maternal SARS-CoV-2 infection and altered intestinal and oral microbial profiles in newborns (Juarez-Castelan et al., 2022; Leftwich et al., 2023). The lack of data highlights the need for further research into the effects of prenatal COVID-19 exposure on neonatal microbiome development.

In this study, our aim was to compare the early gut microbiome composition between infants born to mothers who had COVID-19 during pregnancy and those born to healthy mothers. We focused on bacterial diversity and signs of dysbiosis. Additionally, we sought to explore whether the timing of the SARS-CoV-2 infection in mothers had any impact on the gut microbiome composition in infants. Our healthy controls were recruited before the COVID-19 outbreak, which eliminated the possibility of latent or asymptomatic cases. Furthermore, we applied crucial adjustments, such as gestational age, mode of delivery, and antibiotic use.

## 2 Materials and methods

### 2.1 Study participants

Ninety-four mother-infant pairs (case group) were recruited from the Kulakov Research Centre for Obstetrics, Gynecology, and Perinatology between April and October 2021. The mothers had contracted COVID-19 during pregnancy, as confirmed by standard PCR testing for SARS-CoV-2. The control group consisted of 44 infants recruited before the COVID-19 pandemic, in 2018, who had not been tested for SARS-CoV-2 infection.

### 2.2 Ethical considerations

The study protocol was approved by the local ethics committee of the Kulakov Research Center for Obstetrics, Gynecology, and Perinatology (excerpt from protocol No. 4 from 12.04.2018 and protocol No. 3 from 8.04.2021). All participants provided an informed consent form before enrolment.

### 2.3 Sample collection

Stool samples were collected from women in the case group before delivery upon admission and from infants in both the case and control groups on days 5–7 after birth. The samples were aliquoted into 2-mL cryovials and stored at −80°C until DNA extraction.

### 2.4 DNA extraction

DNA samples were extracted using the QIAamp Fast DNA Stool Mini Kit. The first step of the manufacturer’s instructions was modified to increase DNA yield in the following manner: thawed 180–220 mg stool samples were treated with 600 μL of phenol: chloroform: isoamyl alcohol (25, 24: 1, v/v) and 600 μL of SDS/EDTA buffer solution; the treated samples were mixed with 0.1 mm glass beads at 6,000 rpm for 3 min using the Precellys Evolution Touch Homogenizer (Bertin Technologies, France); the samples were then heated at 70°C for 30 min, centrifuged for 10 min at 12,000 rpm, and the 400 μL supernatant was placed into a fresh tube and mixed by vortexing with 400 μL of the AL buffer solution and 25 μL of proteinase K from provided in the Mini Kit. The next steps followed the manufacturer’s instructions without modifications.

### 2.5 Sequencing

16S rRNA gene libraries were prepared using high-fidelity DNA polymerase from the 2× KAPA HiFi HotStart ReadyMix and V3–V4-specific amplification primers. The 16S Forward Primer was 5′ TCG TCG GCA GCG TCA GAT GTG TAT AAG AGA CAG CCT ACG GGN GGC WGC AG, and the 16S Reverse Primer was 5′ GTC TCG TGG GCT CGG AGA TGT GTA TAA GAG ACA GGA CTA CHV GGG TAT CTA ATC C (Klindworth et al., 2013). The amplification process involved the following steps: 95°C for 3 min; 25 cycles of 95°C for 30 s; 55°C for 30 s; and 72°C for 30 s, followed by 72°C for 5 min; and storage at 4°C.

PCR products were purified using AMPure XP paramagnetic beads. The purified PCR products were then amplified using unique indexes from the Nextera XT Index Kit, following these steps: 95°C for 3 min; 8 cycles of 95°C for 30 s, 55°C for 30 s, and 72°C for 30 s, 72°C for 5 min; storage at 4°C. The lengths of DNA fragments were determined using the Agilent 2100 Bioanalyzer (Agilent Technologies, United States) with Chip DNA 1000 and DNA 1000 High Sensitivity kits, aiming for an amplicon size of approximately 630 bp. The concentration of the DNA library was measured with Qubit 4 (Thermo Fisher Scientific, United States) and the Qubit^™^ 1X dsDNA HS Assay Kits.

The purified libraries were pooled in equal molar ratios, and the PhiX library at 5% was added for quality control. A fresh dilution of 0.2 N NaOH was used to denature the pooled libraries. HT1 hybridization buffer was added to the denatured pool to achieve a final loading concentration of 4 pM and heated in a thermostat at 96°C for 2 min. The MiSeq (Illumina) and MiSeq Reagent Kit v2 (500 cycles) were used for sequencing, with 100,000 reads per sample and pair-end sequencing (251-8-8-251).

### 2.6 Bioinformatic and statistical analysis

A total of 138 infant fecal samples and 94 mother fecal samples were analyzed. Raw DNA sequence reads were processed using the QIIME2 bioinformatics pipeline, with default settings for quality control, chimeric sequence removal, denoising, and data merging. For the mothers’ samples, the median and mean numbers of reads per sample were 90,652 and 87,555, respectively. For the infants’ samples, the median and mean numbers of reads per sample were 65,136 and 65,352, respectively. We prepared alpha rarefaction curves and excluded all samples with a sequencing depth below the plateau level. The minimum sequencing depth was 13,452 and 6,758 for the mothers’ and infants’ samples. Taxonomy assignment was performed using Naive Bayes classifiers trained on Silva 138 99% OTUs full-length sequences (Robeson et al., 2021). The ZymoBIOMICSTM Microbial Community Standard was used as a positive control. After all processing steps, 42.11% of the infant DNA sequence reads and 62.19% of the mother DNA sequence reads remained. Statistical analysis was conducted in an R environment using the phyloseq, vegan, and ANCOMBC packages.

Analysis of Compositions of Microbiomes with Bias Correction 2 (ANCOM-BC2) was used for differential abundance assessment (Lin and Peddada, 2024), with sex, type of delivery, and gestational age, as well as the intake of antibiotics in the third trimester as covariates for infant sample analysis and the intake of antibiotics in different trimesters of pregnancy as a covariate for mother sample analysis.

The Bray–Curtis dissimilarity and UniFrac distances with an NMDS ordination were used for beta-diversity assessment. Significant differences in beta-diversity were tested using a technique described by Anderson et al. (2006), implemented using vegan’s adonis2 function for intergroup dispersions and anova.betadisper, a multivariate analogue of Levene’s test, for group dispersions, with the above covariates.

The Shannon and Chao indices were used for alpha-diversity assessment using ANOVA tests with the above covariates. Tukey’s HSD (Tukey honest significant differences) was used for pairwise comparisons of mother samples.

## 3 Results

### 3.1 Study population

The majority of women from both case and control groups had vaginal delivery; the median gestational age was 39 weeks in both groups (Table 1). Two women from the case group had preterm delivery. Antibiotics before delivery were given to 17.2% of mothers with COVID-19 and 4.5% of healthy mothers.

**Table 1:** Demographic and clinical characteristics of study participants.

Variable		Case group [*n* (%)]	Control group [*n* (%)]	Difference between the case and control groups, *p*
Mothers				
Delivery	Vaginal	79 (84%)	23 (62.2%)	0.001
	Cesarian section	15 (16%)	14 (37.8%)	
Preterm delivery		2 (2.1%)	0 (0%)	1.00
Antibiotics before delivery (90 days before delivery)	Yes	16 (17.2%)	2 (4.5%)	0.06
	No	77 (82.8%)	42 (95.5%)	
COVID-19 exposure				
First trimester		27 (28.7%)	—	—
Second trimester		42 (44.7%)	—	—
Third trimester		25 (26.6%)	—	—
Infants				
Median gestational age, weeks		39 [39; 40]	39 [38; 40]	0.05
Sex	Male	40 (42.6%)	23 (52.3%)	0.36
	Female	54 (57.4%)	21 (47.7%)	
Median Apgar score at 1 min		8 [8; 8]	8 [8; 8]	0.17

### 3.2 Richness, diversity, and composition of infants’ fecal microbiome in case and control groups

**Figure 1:**
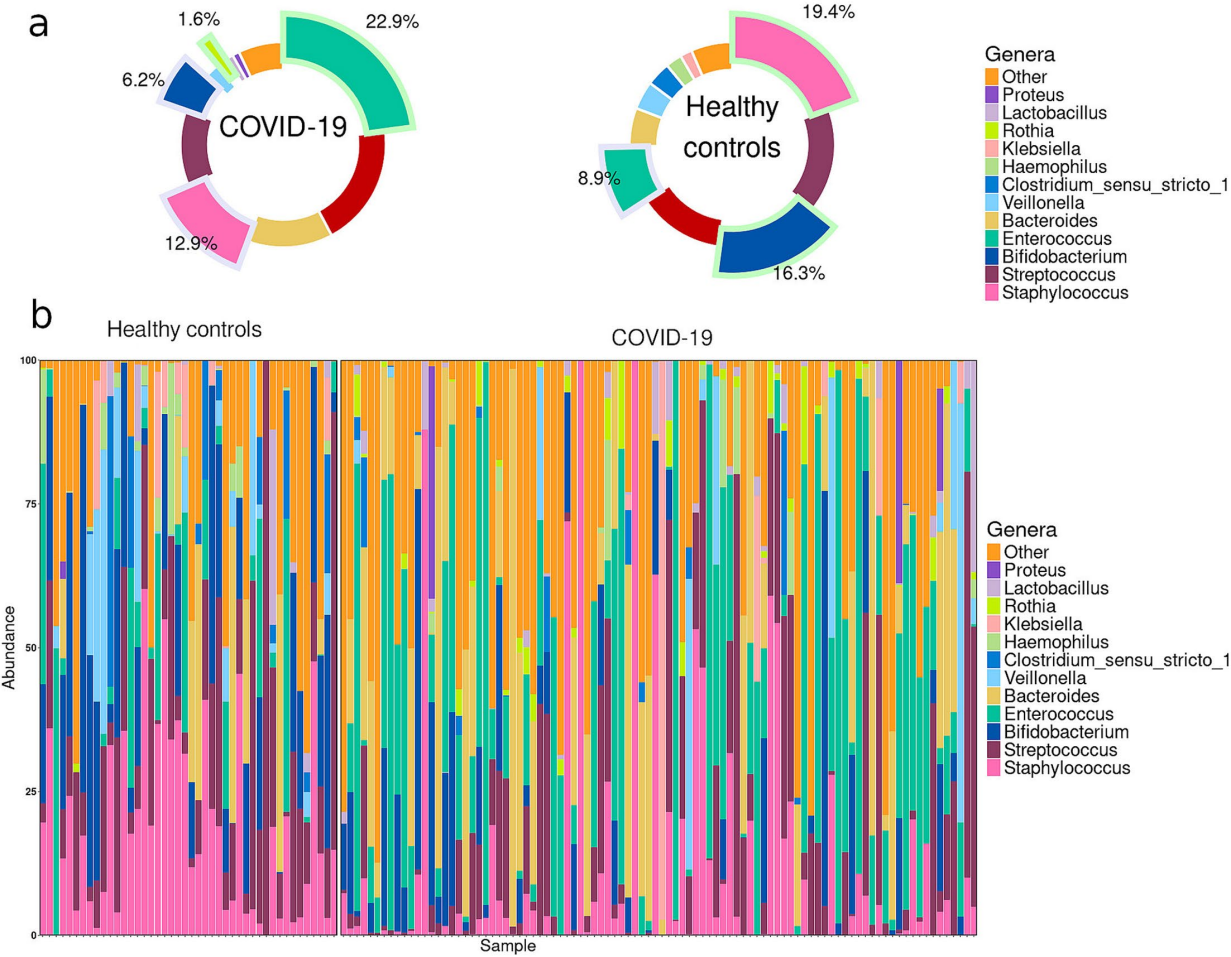
**(a)** Pie charts demonstrating the most prevalent genera in the infant gut microbiome in case and control groups. Slices highlighted with green indicate the genera that were significantly enriched in one of the groups; slices highlighted with blue indicate the genera significantly depleted in one of the groups. **(b)** Abundance plots for individual infant samples in case and control groups.

In the case group, the most prevalent genera in infant fecal samples were *Enterococcus* (22.9%), *Escherichia-Shigella* (19.3%), *Bacteroides* (13.4%), *Staphylococcus* (12.9%), and *Streptococcus* (11.8%), collectively accounting for more than 80% of the fecal microbiome. In the control group, the genera *Staphylococcus* (19.4%), *Streptococcus* (16.4%), *Veillonella* (16.3%), *Escherichia-Shigella* (13.8%), and *Enterococcus* (8.9%) dominated in the infant fecal samples, collectively accounting for more than 70% of the fecal microbiome (Figure 1).

**Figure 2:**
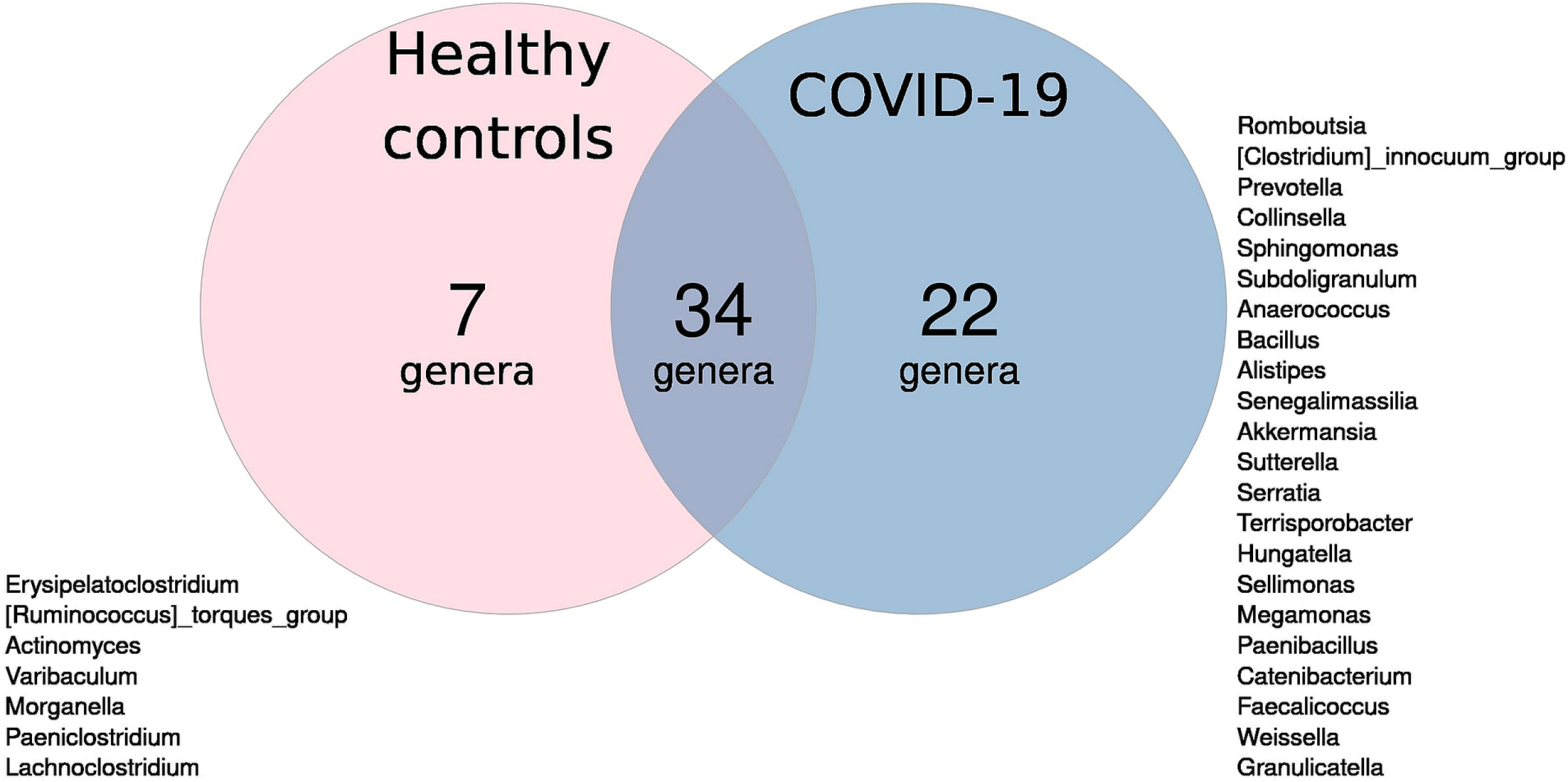
Venn diagram of microbial genera: blue circle—unique to infants in case group; pink circle—unique to infants in control group; overlap—found in both groups.

Thirty-four genera were detected in infant fecal samples in both groups. Seven genera were unique to the case group, while 22 genera were unique to the control group (Figure 2).

**Figure 3:**
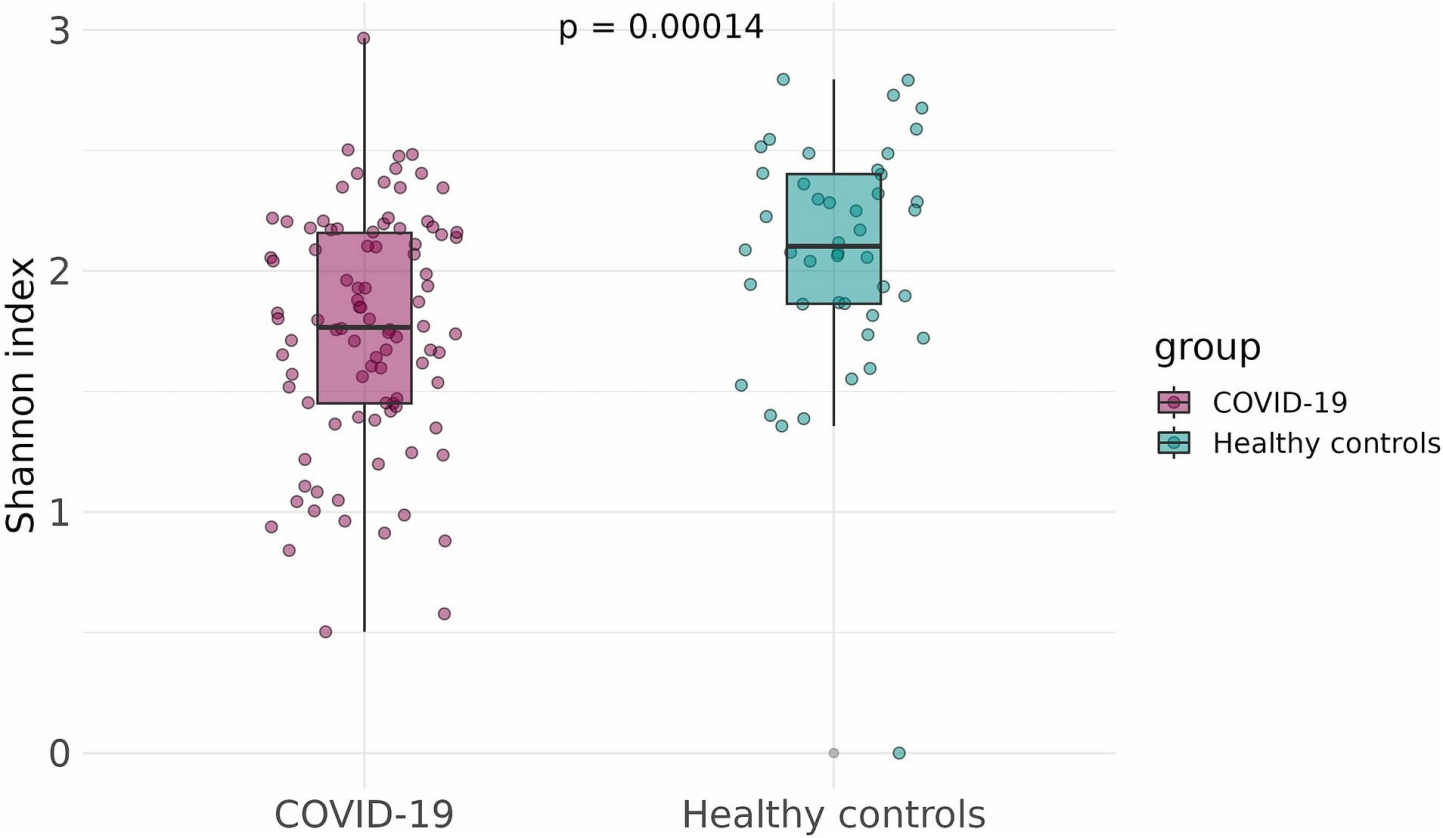
Shannon indices of infant fecal microbiome in case and control groups.

**Figure 4:**
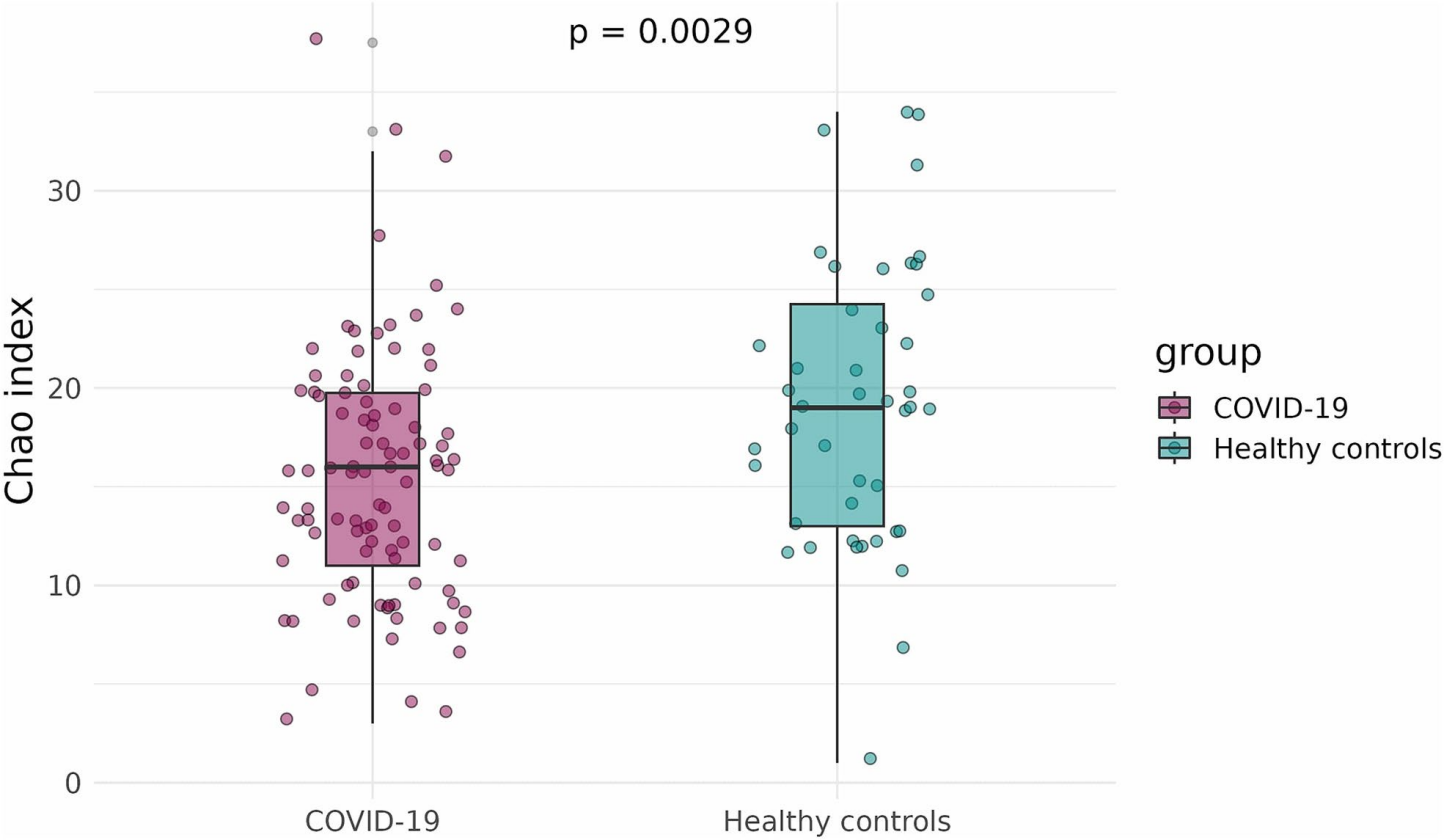
Chao indices of infant fecal microbiome in case and control groups.

To compare the alpha-diversity of the infant fecal microbiome in case and control groups, the Shannon and Chao indices were calculated and adjusted for infants’ sex, gestational age, mode of delivery, and antibiotics intake. The Shannon and Chao indices were significantly lower in infants that had been exposed to COVID-19 in the womb (*p* = 1.5 × 10^−5^ and *p* = 0.0029, respectively) (Figures 3, 4).

**Figure 5:**
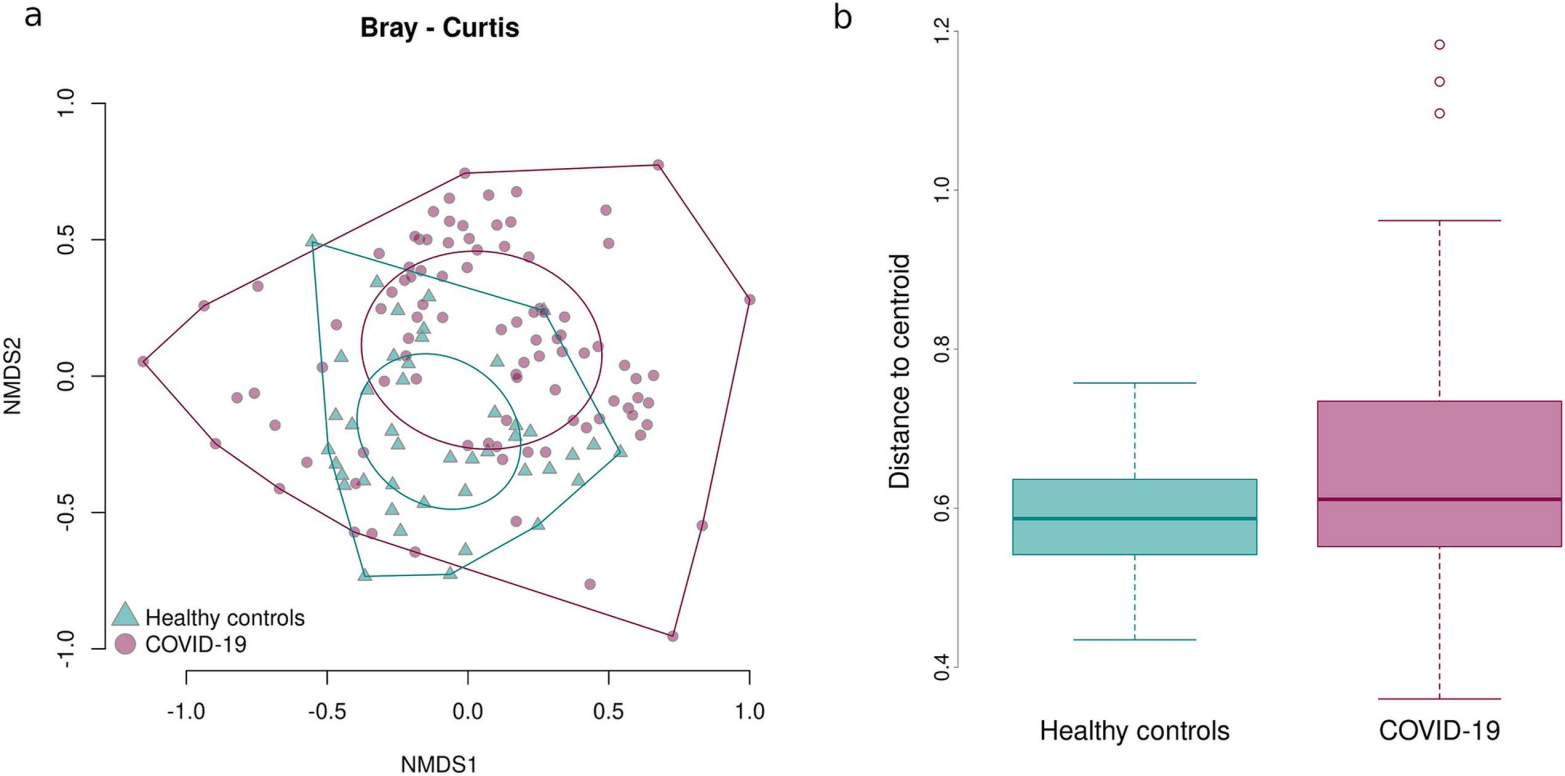
Bray–Curtis dissimilarity between the samples in case and control groups. Sample dispersion in case and control groups. **(a)** Graph showing distances between the center of the oval and individual samples. **(b)** Boxplot showing average distances between the center of the oval and individual samples.

**Figure 6:**
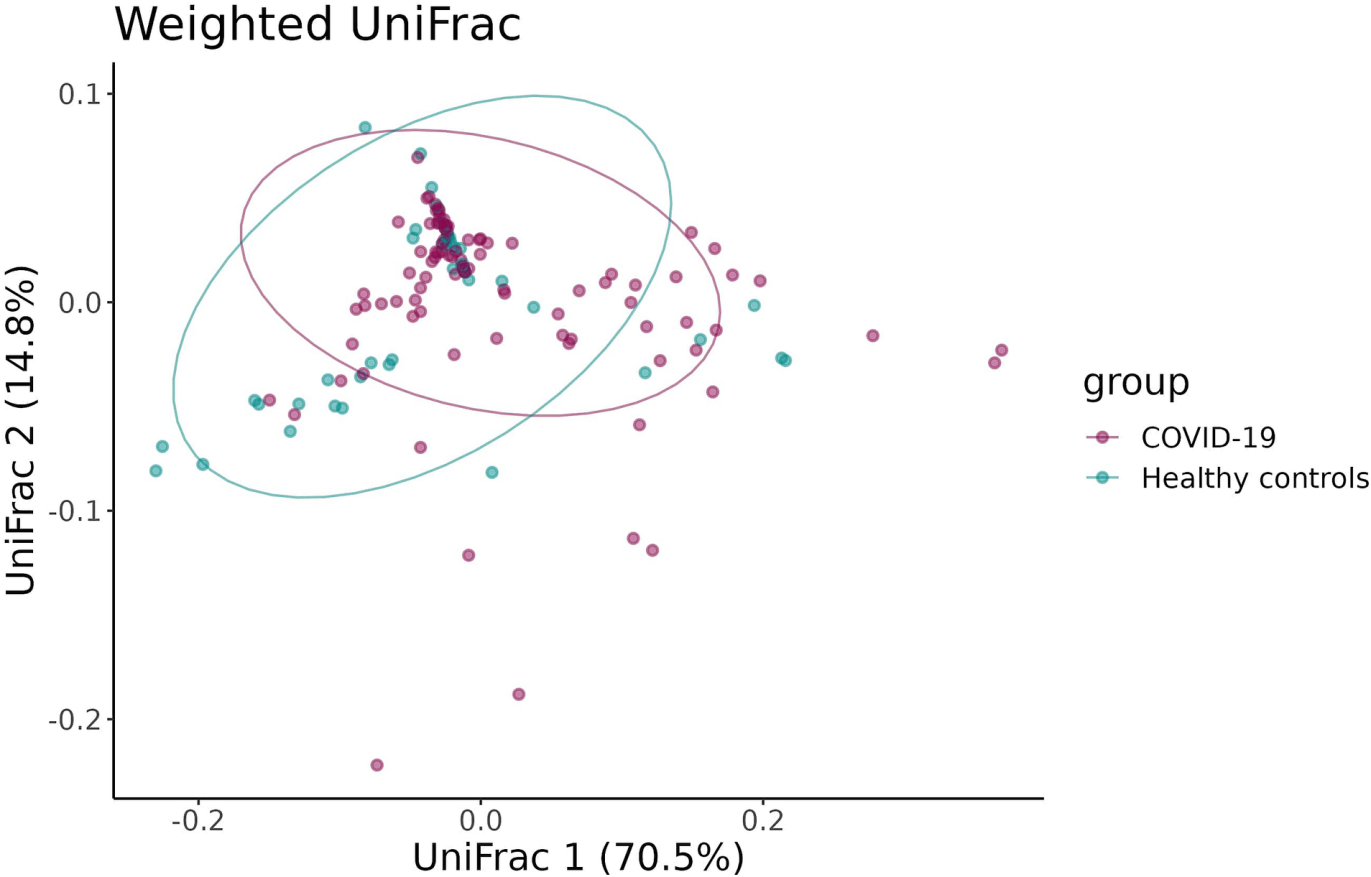
UniFrac distance between the samples in case and control groups.

To estimate the beta-diversity of infant fecal samples in the case and control groups, the UniFrac distance and the Bray–Curtis dissimilarity were calculated and adjusted for infants’ sex, gestational age, mode of delivery, and antibiotic intake. Both the UniFrac distance and the Bray–Curtis dissimilarity revealed distinct clustering of samples in the groups (*p* = 0.02 and *p* = 0.03, respectively) (Figures 5a, 6).

The Bray–Curtis dissimilarity showed that the fecal samples of infants in the case group significantly differed from one another, while the fecal samples of infants born to healthy mothers tended to be more similar, as shown by the larger oval in Figure 5a encompassing the fecal samples from the case group. This indicates a greater inter-sample variability of the fecal microbiome composition in the case groups and a greater similarity of the fecal microbiome composition in the control group.

To confirm the detected inter-sample variability in the case group, sample dispersion was assessed in both groups: the distance between the center of the respective oval and each point representing an individual sample was measured and the average distances were calculated and compared. Fecal samples of infants prenatally exposed to COVID-19 demonstrated a significantly higher dispersion, i.e., variability in the microbial composition, than fecal samples of infants that had not been prenatally exposed to COVID-19 (*p* = 0.03211) (Figure 5b).

**Figure 7:**
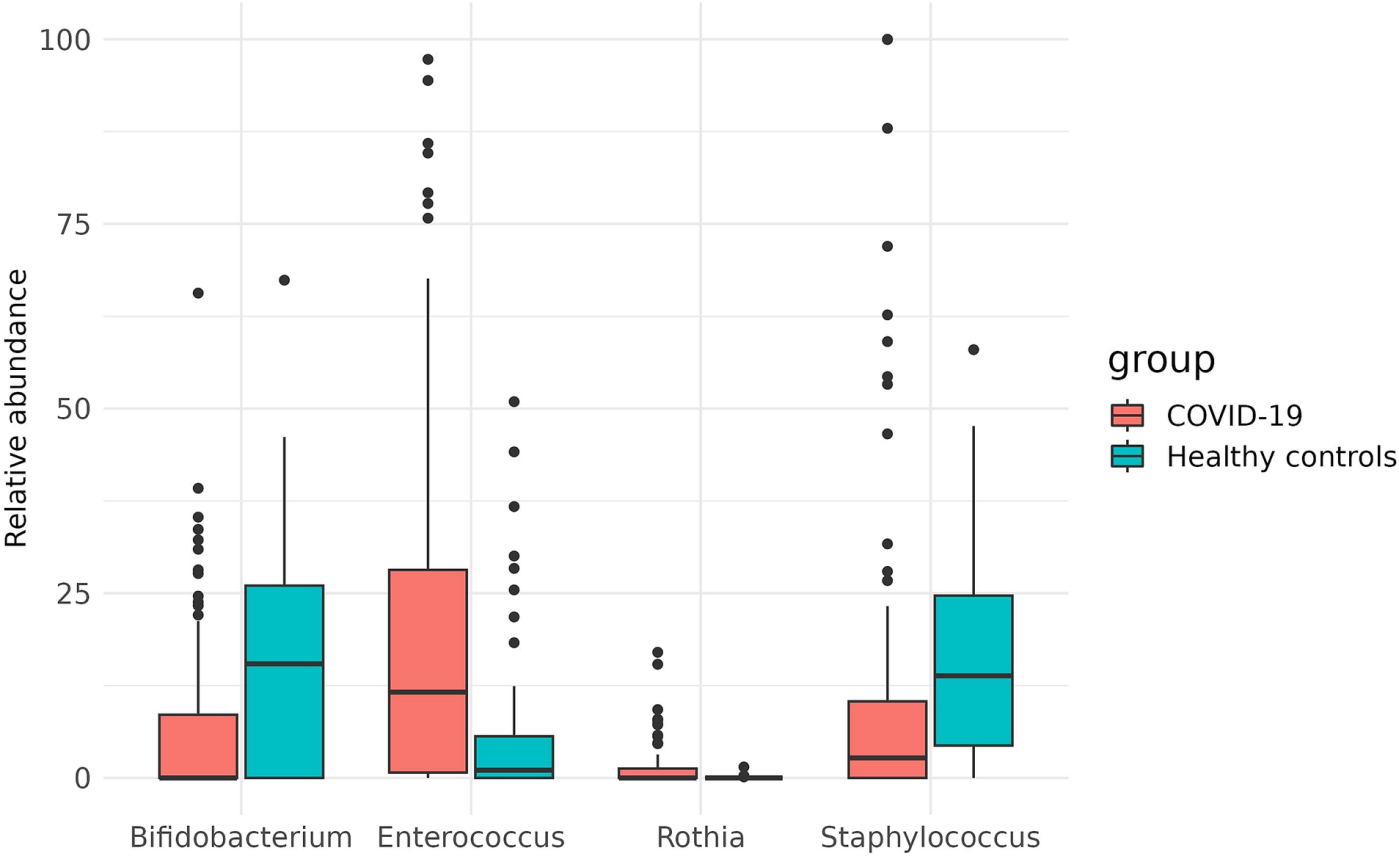
Differences in the abundance of taxons in case and control groups.

Additionally, the two groups were compared for the abundance of all taxonomic groups detected. In the case group, *Bifidobacterium* and *Staphylococcus were* significantly depleted (*p* = 0.0049 and *p* = 0.0295, respectively), while the abundance of *Rothia* and *Enterococcus* was increased in this group (*p* = 0.0026 and *p* = 0.0049, respectively) (Figure 7).

### 3.3 Effects of COVID-19 exposure on infant fecal microbiome in different trimesters of pregnancy

**Figure 8:**
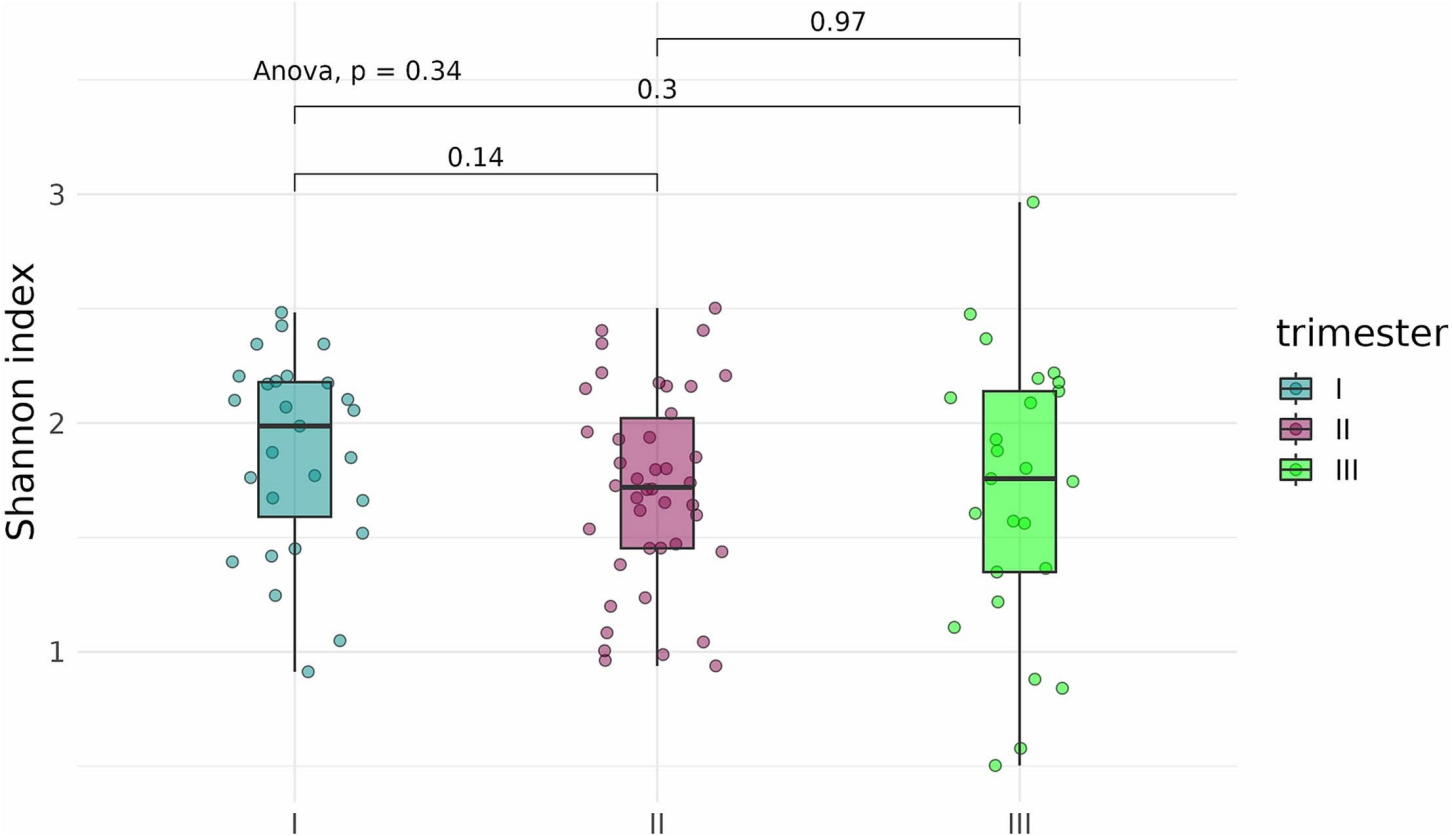
Shannon indices of the fecal microbiome in infants prenatally exposed to COVID-19 in the first, second, or third trimester.

**Figure 9:**
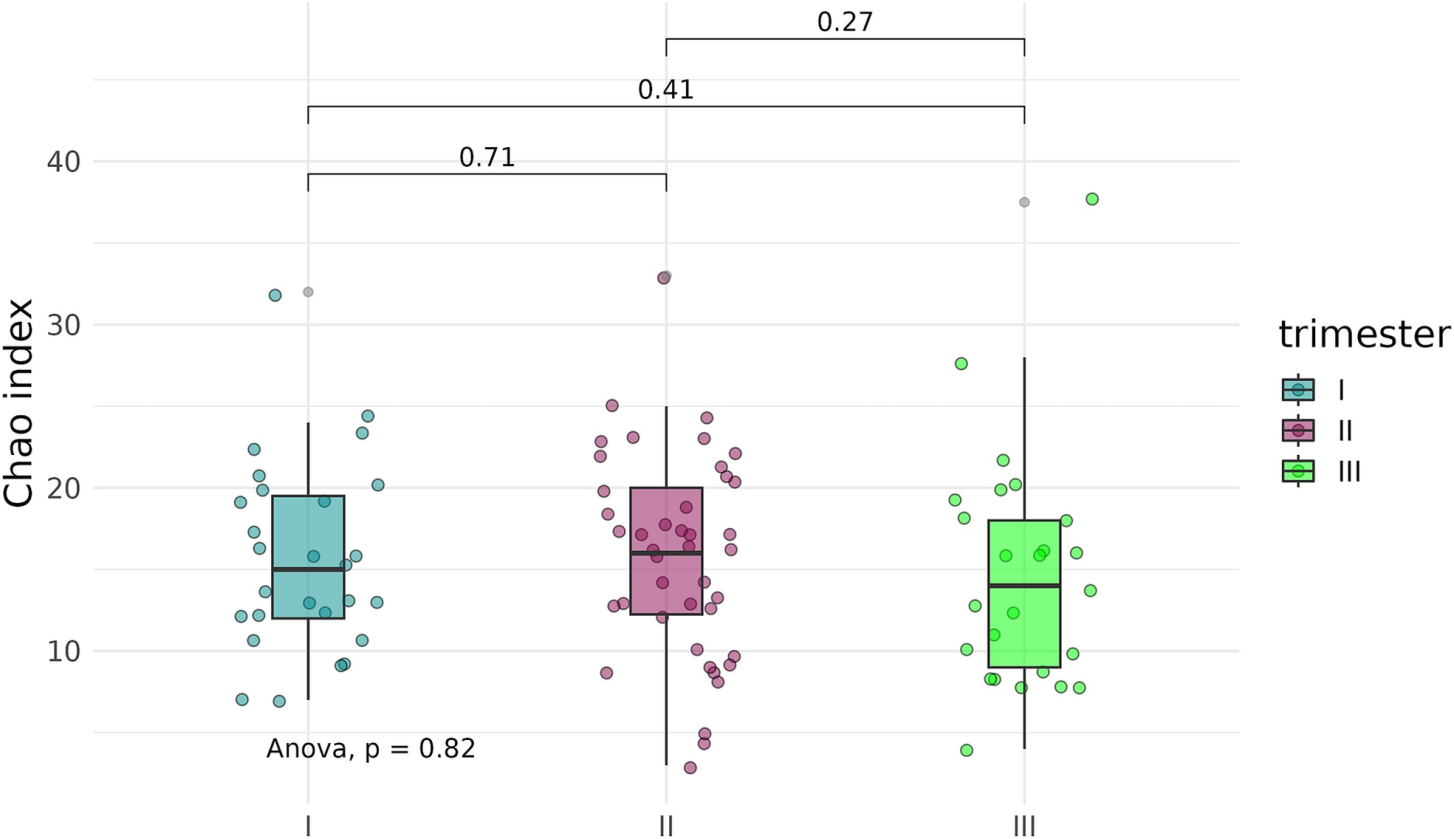
Chao indices of the fecal microbiome in infants prenatally exposed to COVID-19 in the first, second, or third trimester.

To determine whether the timing, or trimester, of prenatal COVID-19 exposure had an effect on the infant gut microbiome, fecal samples of infants exposed to COVID-19 in the first, second, or third trimesters were compared based on alpha-diversity. Neither Shannon indices nor Chao indices differed significantly between the three groups (Figures 8, 9).

**Figure 10:**
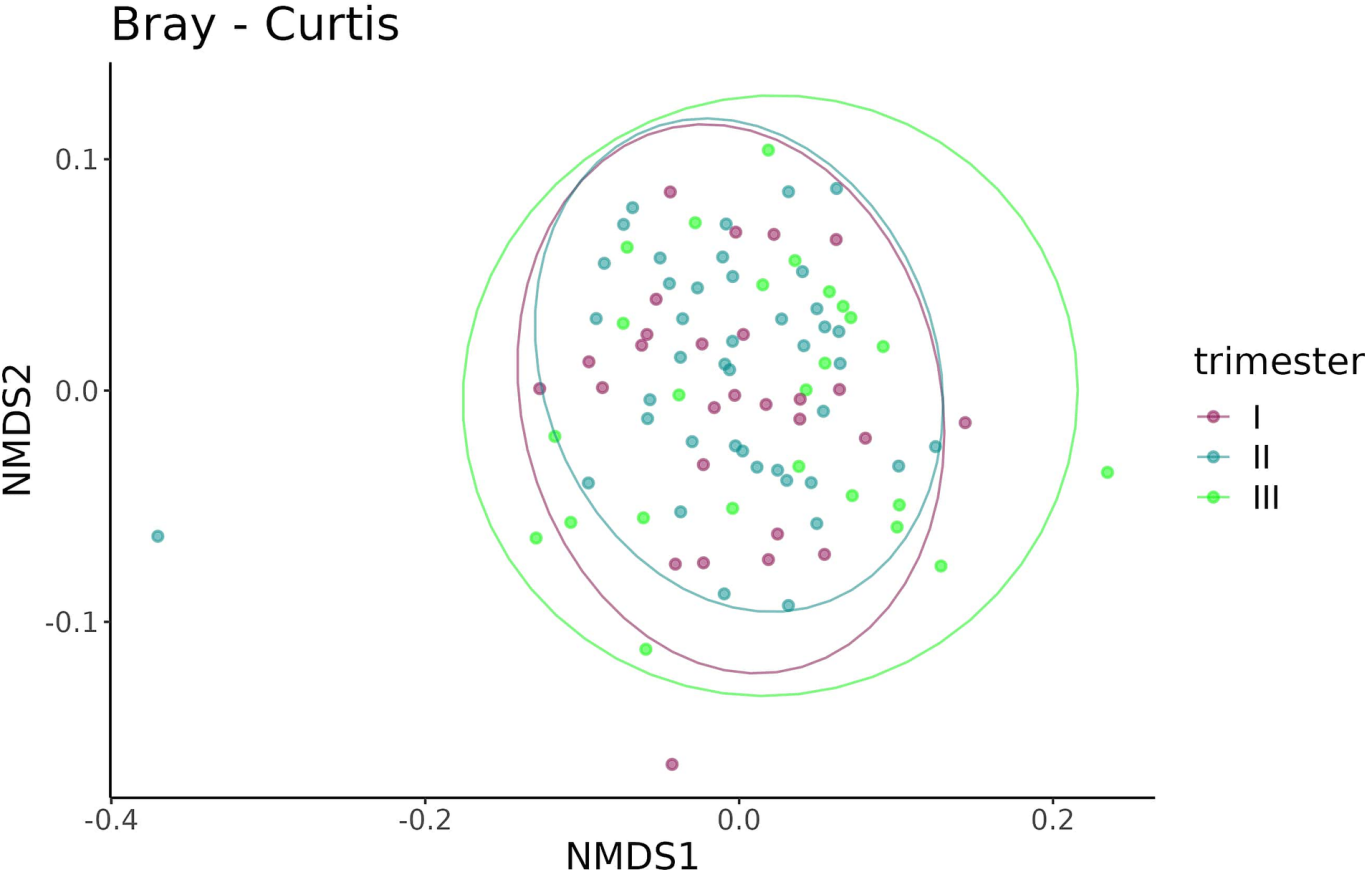
Bray–Curtis dissimilarity between the samples from infants prenatally exposed to COVID-19 in the first, second, or third trimester.

**Figure 11:**
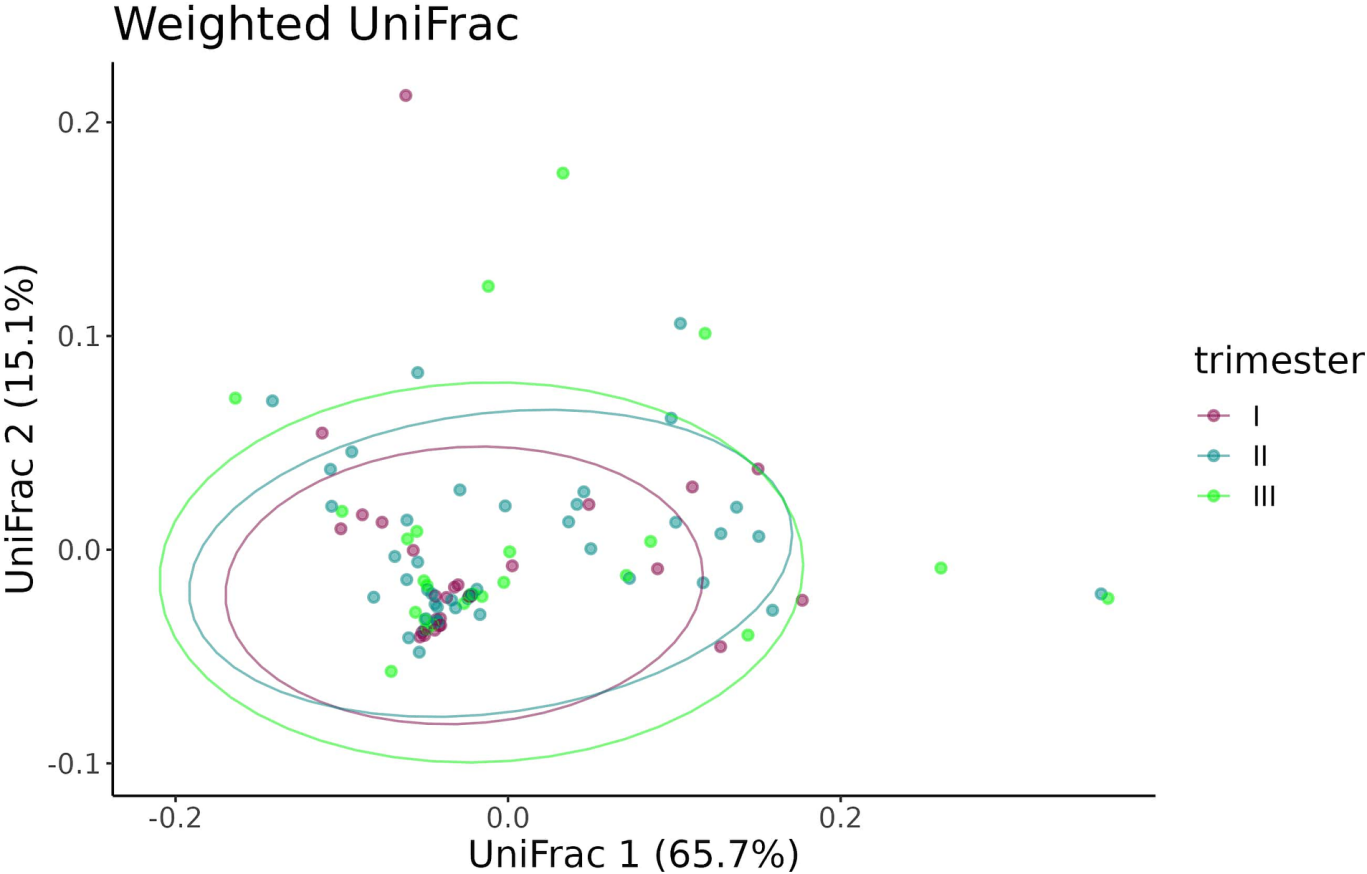
UniFrac distance between the samples from infants prenatally exposed to COVID-19 in the first, second, or third trimester.

A beta-diversity analysis indicated that the trimester of prenatal COVID-19 exposure did not have any effect on the infant gut microbiome, as shown in Figures 10, 11 (*p* = 0.11 for the Bray–Curtis dissimilarity and *p* = 0.87 for the UniFrac distance).

The timing of prenatal COVID-19 exposure did not significantly affect the taxonomic composition of the gut microbiome in infants. No significant differences in taxon abundance were found.

### 3.4 Effect of the timing of COVID-19 contraction on expecting women in different trimesters of pregnancy

**Figure 12:**
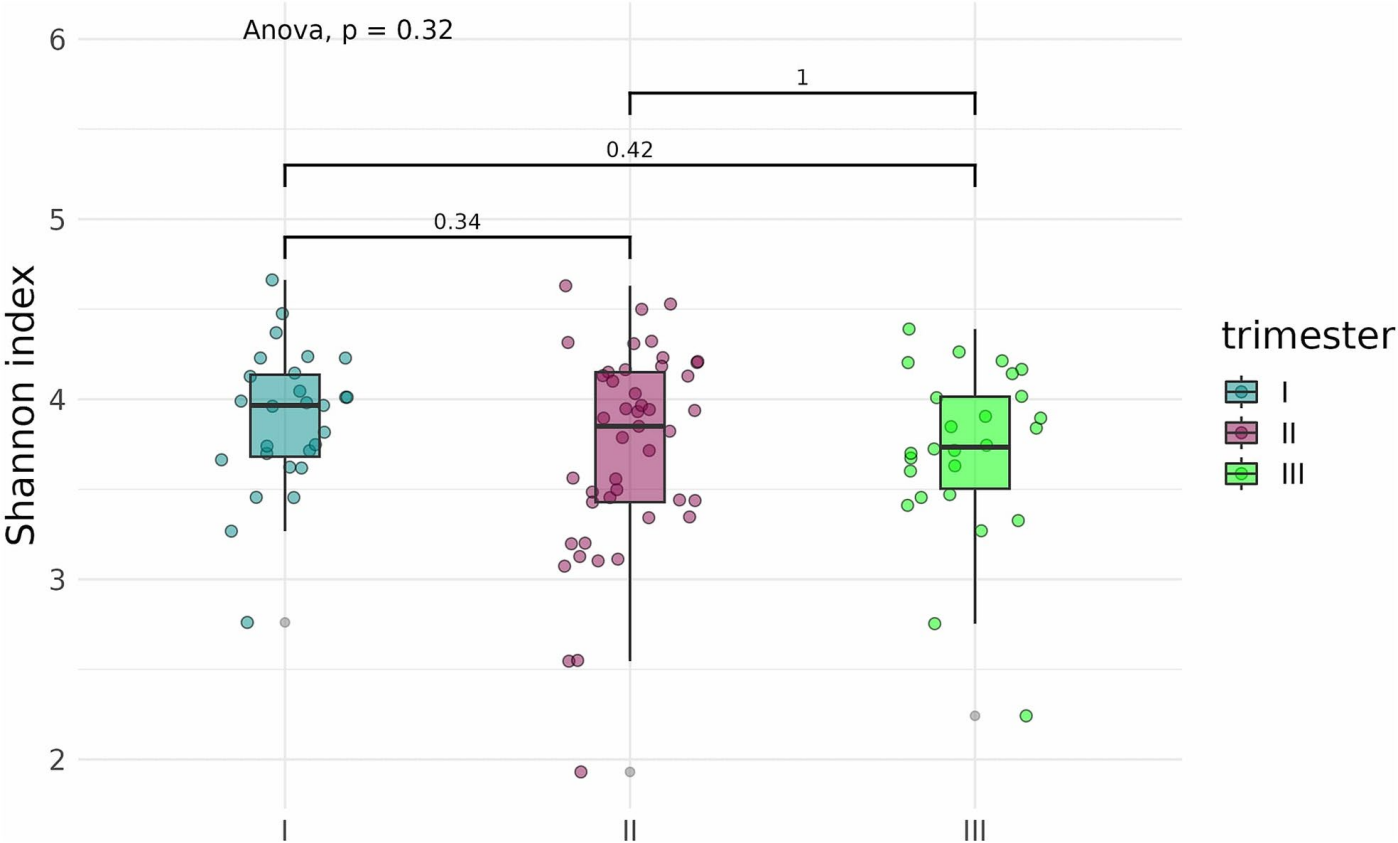
Shannon indices of fecal samples from pregnant women depending on the timing of COVID-19 contraction.

**Figure 13:**
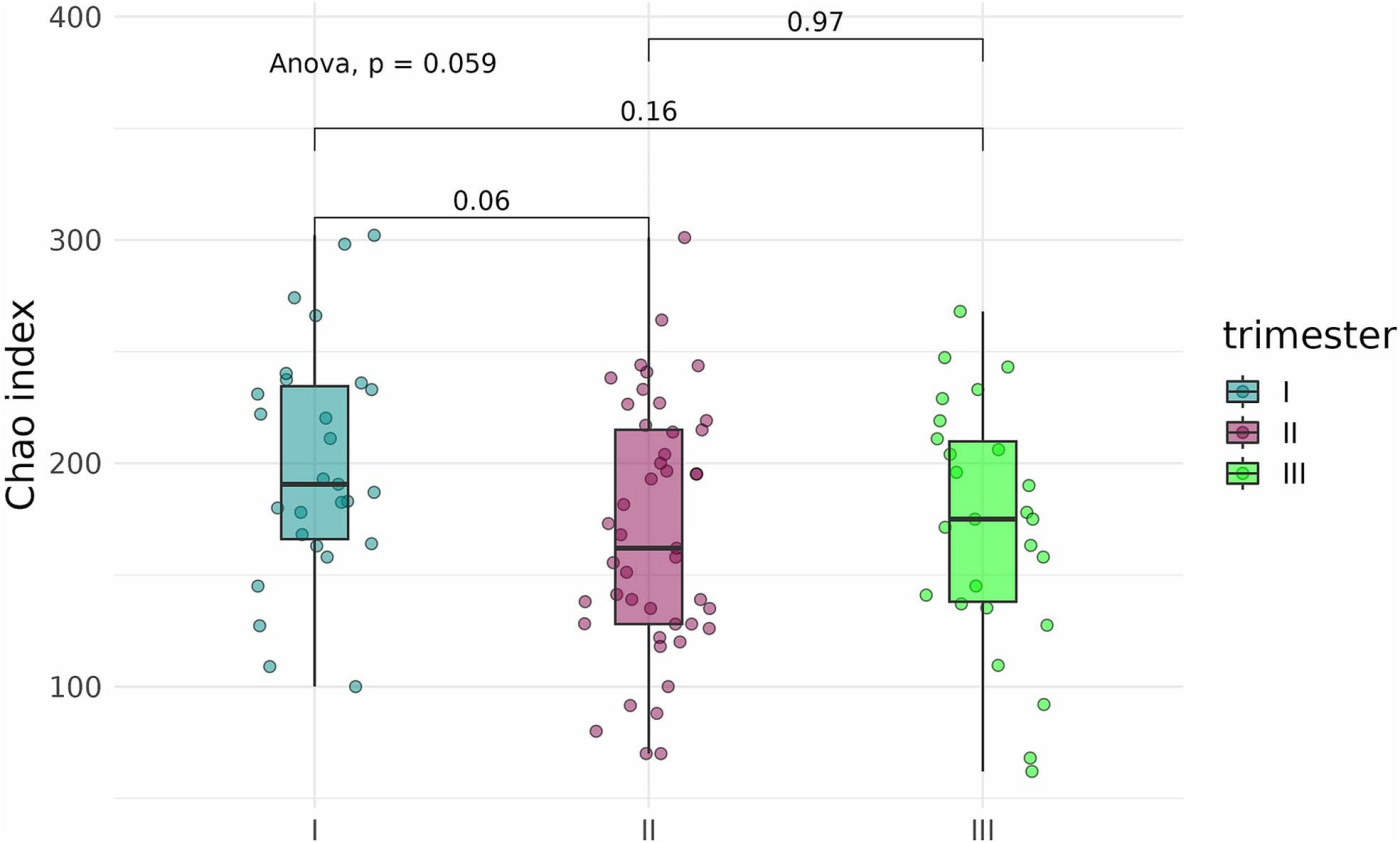
Chao indices of fecal samples from pregnant women depending on the timing of COVID-19 contraction.

An analysis of alpha diversity revealed that the Shannon and Chao indices of fecal samples collected before delivery from pregnant women who contracted COVID-19 in the second (*n* = 42) and third (*n* = 25) trimesters were generally lower than the indices of fecal samples from pregnant women who contracted the infection in the first trimester (*n* = 27) (Figures 12, 13). However, the differences did not reach statistical significance. Comparison of Shannon and Chao indices across the three groups using ANOVA revealed no significant differences (*p* = 0.32 and *p* = 0.059, respectively). In general, we can conclude that contracting COVID-19 in the second and third trimesters tended to produce a more pronounced impact on the fecal microbiome of pregnant women.

**Figure 14:**
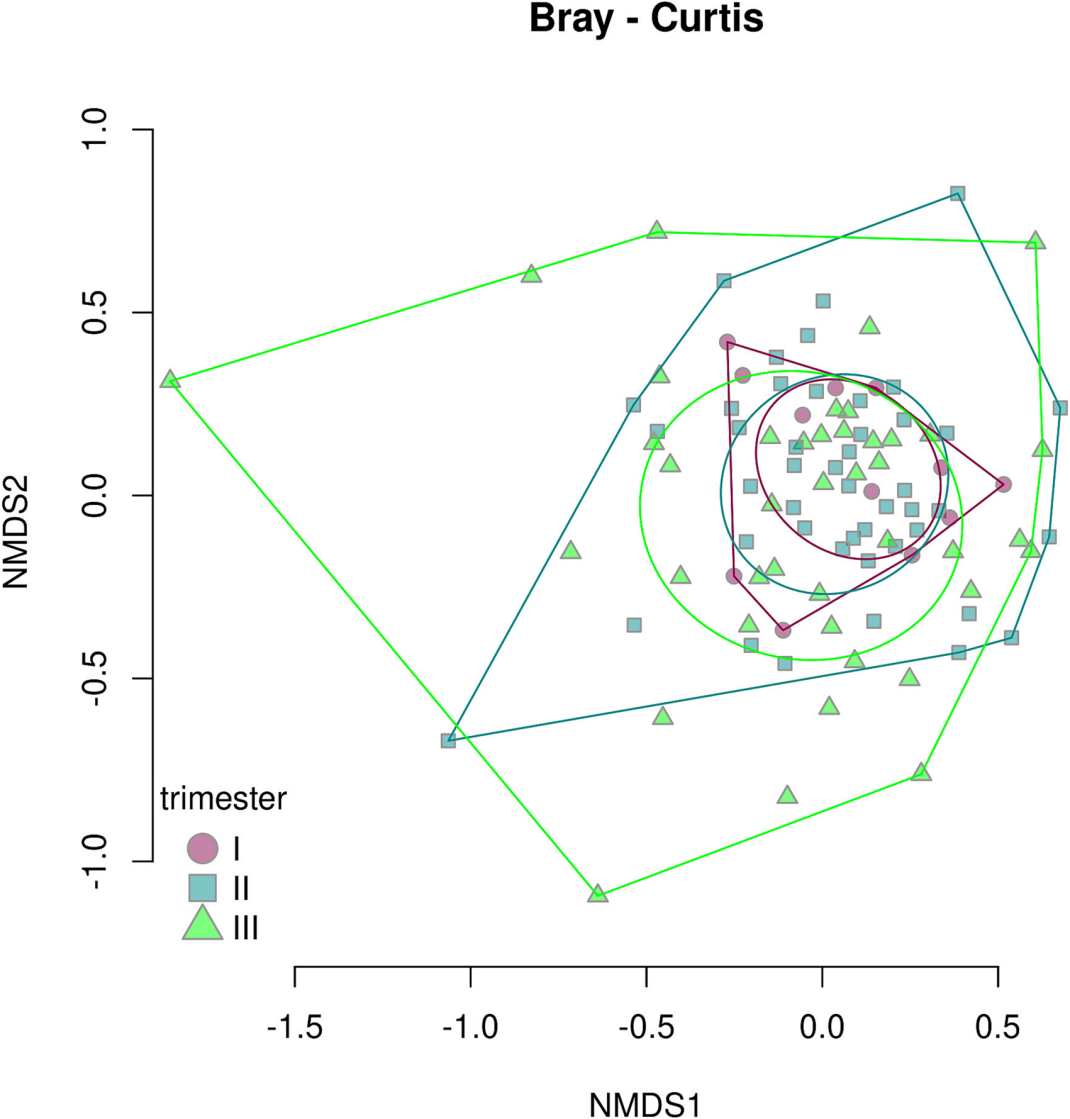
Bray–Curtis dissimilarity between fecal samples from pregnant women depending on the timing of COVID-19 contraction.

**Figure 15:**
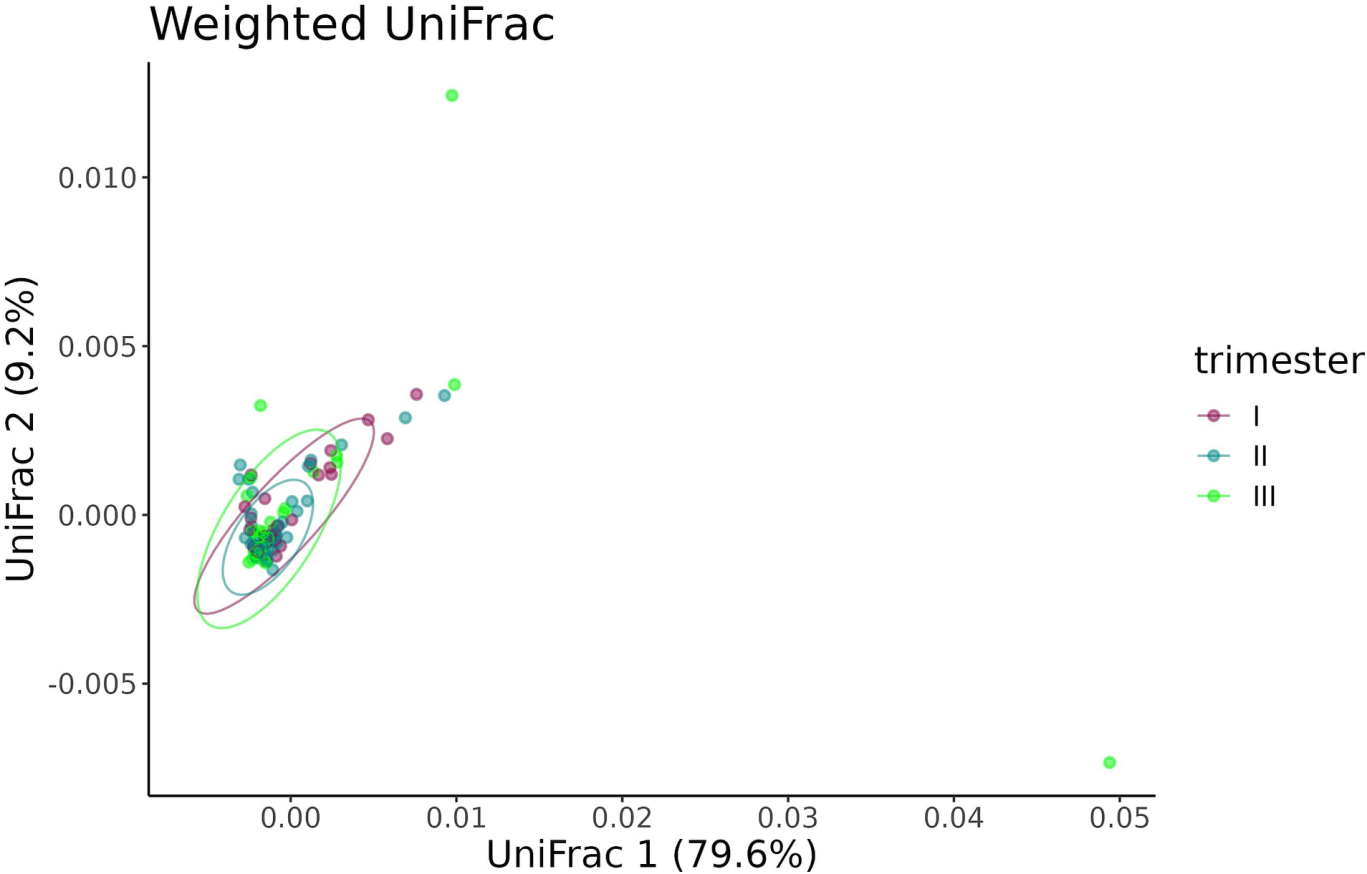
UniFrac distance between fecal samples from pregnant women depending on the timing of COVID-19 contraction.

The Bray–Curtis dissimilarity (Figure 14) did not show a significant difference between fecal samples from pregnant women who contracted COVID-19 in the first, second, or third trimester (*p* = 0.284), whereas the UniFrac analysis revealed distinct clustering of samples depending on the timing of contraction (*p* = 0.049) (Figure 15).

The pairwise comparison of dispersion between the three groups depending on the timing of COVID-19 contraction showed significant difference between the second and third (*p* = 0.035), as well as first and third trimesters (*p* = 0.028), while the first and the second trimesters demonstrated no difference (*p* = 0.950). The highest dispersion was observed in samples obtained from women with recent COVID-19, i.e., those who contracted infection in the third trimester.

## 4 Discussion

We found that SARS-CoV-2 infection during pregnancy significantly affects the initial gut colonization in infants, likely through its impact on the mother’s microbiome. We observed a distinct clustering of fecal samples from infants born to mothers who had COVID-19 during pregnancy (the case group) and those born to healthy mothers (the control group). Additionally, infants in the case group showed a marked reduction in bacterial diversity and richness compared to infants in the control group, as indicated by lower Shannon and Chao indices.

Notably, the control group showed only seven unique genera, despite having higher alpha-diversity compared to the case group, which exhibited 22 unique genera. This finding may indicate that compromised microbiomes, such as those affected by COVID-19, are more prone to stochastic changes, including the acquisition of new microbial species, while healthy microbiomes tend to be more stable. We hypothesize that a greater number of unique taxa may indicate dysbiosis rather than increased microbial diversity. The sample dispersion analysis confirmed this hypothesis: infants in the case group showed significantly higher inter-sample variability than infants in the control group.

This finding could be explained by a principle that has been introduced by Zaneveld et al. (2017) who followed a long-standing tradition of literary allusions in scientific terminology—the Anna Karenina principle (AKP). The authors based their term on the opening line of Leo Tolstoy’s novel Anna Karenina, stating that *happy families are all alike; every unhappy family is unhappy in its own way*. Thus, with their novel terminology, the authors conjured up a vivid image of healthy and distressed human microbiomes, the former being alike while the latter being uniquely affected, depending on the cause. In our study, a higher number of unique genera and a wider dispersion of samples in the case group strongly suggest the destabilizing effect of COVID-19 on the human microbiome. A very similar impact of SARS-CoV-2 infection was observed in the upper respiratory tract (URT) microbiome in patients hospitalized with COVID-19 (Ignatyeva et al., 2024). Patients with mild to severe COVID-19 tend to have a less diverse and more unstable URT microbiome. Our findings indicate that the impact of SARS-CoV-2 extends beyond the infected person: infection during pregnancy not only causes changes in the microbiome of a pregnant woman but also affects the early colonization of the infant microbiome.

Based on the available information, only two studies have explored the effects of maternal COVID-19 on infants’ microbiomes. Leftwich et al. (2023) found no association between maternal COVID-19 and any marked changes in the fecal microbiomes of neonates, but they did find significant alterations in their oral microbiome. Juarez-Castelan et al. (2022) tested neonatal rectal swabs and observed a trend towards a higher number of observed species and alpha-diversity in infants prenatally exposed to SARS-CoV-2. Notably, both of the aforementioned studies reported that maternal microbiomes, including oral, gut, and vaginal, in COVID-19 affected groups differed dramatically from those in healthy controls. Several other studies have also described distinct microbial profiles in pregnant women infected with SARS-CoV-2. Crovetto et al. (2022) analyzed the nasopharyngeal microbiome in SARS-CoV-2-infected pregnant women and found changes in both alpha- and beta-diversity, as well as a differential abundance of 18 genera. Celik et al. (2023) demonstrated that COVID-19 in pregnant women resulted in a significantly altered vaginal microbiome.

Notably, the findings on changes in the maternal microbiome and their interpretations have often been conflicting, particularly those regarding alpha-diversity. Leftwich et al. (2023) observed a COVID-19-associated depletion of microbial richness and diversity in all biotopes studied and interpreted it as a marker of dysbiosis. The rest of the above studies, however, reported higher alpha-diversity indices in women who had COVID-19 during pregnancy and interpreted their finding as a marker of dysbiosis. Lower microbial richness and diversity are commonly interpreted as signs of dysbiosis. However, there is evidence to suggest that increased alpha-diversity may be indicative of an unstable microbiome, spontaneously acquiring new microbial species, non-typical of a healthy microbiome (Celik et al., 2023). These conflicting findings may be due to a variety of factors, such as differences in study designs, sequencing methods, data processing algorithms.

In our study, the microbiomes of infants prenatally exposed to COVID-19 demonstrated reduced microbial diversity and richness. However, the higher dispersion of infant fecal samples and a larger number of unique taxa in the case group clearly indicated destabilization. The lower microbial diversity and richness, along with the higher number of unique taxa, may appear to contradict each other. However, these two seemingly conflicting findings illustrate the Anna Karenina principle: compromised microbiomes are less alike and more unique compared to healthy ones. This principle may also explain the link between higher alpha-diversity and disrupted microbiomes observed in other studies.

Therefore, conventional metrics such as alpha- and beta-diversity may not always accurately reflect the condition of the microbiome and could even be misleading. Hence, multiple characteristics of commensal microbial communities, sometimes not obvious, should always be taken into consideration when assessing microbiomes for dysbiosis.

Notably, the timing of SARS-CoV-2 infection during pregnancy appears to have no particular impact on the gut microbiota of infants, as neither alpha-diversity nor beta-diversity varied significantly between infant microbiomes in the three groups depending on the trimester when the mother was infected with SARS-CoV-2.

We expected that contracting COVID-19 shortly before delivery (in the third trimester) would likely leave “fresh” traces in the maternal microbiome, thereby causing more distinct dysbiosis in the infant. However, this hypothesis was confirmed only for mothers who demonstrated more pronounced signs of dysbiosis (such as lower Shannon and Chao indices along with higher dispersion of samples) if they contracted COVID-19 in the second or third trimester as opposed to those who had COVID-19 in the first trimester. This effect, nevertheless, was not extended on their offspring. The microbiomes of infants born to mothers with “old” traces of COVID-19 dating back to the first trimester were indistinguishable from those of infants born to mothers who had COVID-19 later in their pregnancy, whose microbiomes had less time for its normalization.

Our results clearly indicate the ability of COVID-19 to produce long-lasting negative effects on both affected women and their infants, which is particularly concerning given the indisputable importance of the early-life microbiome in child development. Being the main source of the primary microbial colonization that starts during the delivery, the maternal microbiome is essential for the establishment of the infant’s microbiome. However, growing evidence suggests that the effect of the maternal microbiome is not limited to peri- and post-natal transfer of commensal microorganisms from the mother to the child. Husso et al. (2023) demonstrated that the maternal microbiome is a crucial factor that can significantly modulate fetal development by affecting its gene expression via microbial metabolites. Among all organs studied, the fetal intestine was found to be affected the most by the maternal microbiome, which manifested as altered expression levels of critical genes responsible for the commensal microbiota tolerance, innate immune reactions, and epithelial barrier maintenance (Husso et al., 2023). SARS-CoV-2 during pregnancy can disrupt the expression of tight-junction proteins in the mother, thus impairing normal barrier function in the gut (Tsounis et al., 2023), induce oxidative stress in the epithelium and cause its damage (Georgieva et al., 2023; Berber et al., 2024), as well as trigger inflammation. These processes can violate a normal crosstalk between the mother’s microbiome and the fetus, which can potentially affect its normal development.

Further studies, particularly longitudinal ones, would offer a better understanding of the mechanisms underlying the negative impact of COVID-19 and its persistent effects on the microbiome. Probiotic agents have demonstrated their effectiveness in restoring COVID-19-associated gut dysbiosis (Zhang et al., 2022). Therefore, these agents may currently be one of the most effective ways to normalize infant microbiomes compromised by prenatal COVID-19 exposure.

There are few limitations to our findings, some of which are typical of a cross-sectional study. Since the control group was recruited before the COVID-19 outbreak, mothers’ samples were not collected, and changes in their microbiomes were not tracked. The clinical characteristics of mother-infant pairs were not assessed after discharge. A follow-up would have provided valuable information. Therefore, additional studies are needed to examine the effects of prenatal COVID-19 exposure on early microbiome establishment and overall health in infants.

The validity of our findings is supported by two crucial factors. Healthy controls for our study were recruited before the COVID-19 outbreak, thereby eliminating any probability of latent or asymptomatic infection in the control group. All the presented results were adjusted for the most important covariates, minimizing the risk of bias. Thus, it is safe to conclude that our findings accurately demonstrate the true effect of COVID-19 on both pregnant women and their infants.

## Data Availability

The datasets presented in this article are not readily available because the institution that provided us with sequencing services adheres to stringent data security protocols that restrict public data sharing. However, the data can be made available upon request, requests to access the datasets should be directed to the corresponding author.

## References

[ref1] Anderson M. J. Ellingsen K. E. Mcardle B. H. (2006). Multivariate dispersion as a measure of beta diversity. Ecol. Lett. 9, 683–693. doi: 10.1111/j.1461-0248.2006.00926.x, PMID: 16706913

[ref2] Berber N. K. Kurt O. Altintop Geckil A. Erdem M. Kiran T. R. Otlu O.. (2024). Evaluation of oxidative stress and endothelial dysfunction in COVID-19 patients. Medicina 60:1041. doi: 10.3390/medicina60071041, PMID: 39064471 PMC11279166

[ref3] Browne H. P. Shao Y. Lawley T. D. (2022). Mother-infant transmission of human microbiota. Curr. Opin. Microbiol. 69:102173. doi: 10.1016/j.mib.2022.102173, PMID: 35785616

[ref4] Celik E. Ozcan G. Vatansever C. Paerhati E. Kuskucu M. A. Dogan O.. (2023). Alterations in vaginal microbiota among pregnant women with COVID-19. J. Med. Virol. 95:e28132. doi: 10.1002/jmv.28132, PMID: 36068653 PMC9538183

[ref5] Crovetto F. Selma-Royo M. Crispi F. Carbonetto B. Pascal R. Larroya M.. (2022). Nasopharyngeal microbiota profiling of pregnant women with SARS-CoV-2 infection. Sci. Rep. 12:13404. doi: 10.1038/s41598-022-17542-z, PMID: 35927569 PMC9352760

[ref6] Georgieva E. Ananiev J. Yovchev Y. Arabadzhiev G. Abrashev H. Abrasheva D.. (2023). COVID-19 complications: oxidative stress, inflammation, and mitochondrial and endothelial dysfunction. Int. J. Mol. Sci. 24:14876. doi: 10.3390/ijms241914876, PMID: 37834324 PMC10573237

[ref7] Husso A. Pessa-Morikawa T. Koistinen V. M. Karkkainen O. Kwon H. N. Lahti L.. (2023). Impacts of maternal microbiota and microbial metabolites on fetal intestine, brain, and placenta. BMC Biol. 21:207. doi: 10.1186/s12915-023-01709-9, PMID: 37794486 PMC10552303

[ref8] Ignatyeva O. Gostev V. Taraskina A. Tsvetkova I. Pavlova P. Sulian O.. (2024). General dynamics of the URT microbiome and microbial signs of recovery in COVID-19 patients. Benef. Microbes 15, 145–164. doi: 10.1163/18762891-bja00004, PMID: 38412868

[ref9] Juarez-Castelan C. J. Velez-Ixta J. M. Corona-Cervantes K. Pina-Escobedo A. Cruz-Narvaez Y. Hinojosa-Velasco A.. (2022). The entero-mammary pathway and perinatal transmission of gut microbiota and SARS-CoV-2. Int. J. Mol. Sci. 23:10306. doi: 10.3390/ijms231810306, PMID: 36142219 PMC9499685

[ref10] Kapourchali F. R. Cresci G. A. M. (2020). Early-life gut microbiome-the importance of maternal and infant factors in its establishment. Nutr. Clin. Pract. 35, 386–405. doi: 10.1002/ncp.10490, PMID: 32329544

[ref11] Klindworth A. Pruesse E. Schweer T. Peplies J. Quast C. Horn M.. (2013). Evaluation of general 16S ribosomal RNA gene PCR primers for classical and next-generation sequencing-based diversity studies. Nucleic Acids Res. 41:e1. doi: 10.1093/nar/gks808, PMID: 22933715 PMC3592464

[ref12] Leftwich H. K. Vargas-Robles D. Rojas-Correa M. Yap Y. R. Bhattarai S. Ward D. V.. (2023). The microbiota of pregnant women with SARS-CoV-2 and their infants. Microbiome 11:141. doi: 10.1186/s40168-023-01577-z, PMID: 37365606 PMC10291758

[ref13] Lin H. Peddada S. D. (2024). Multigroup analysis of compositions of microbiomes with covariate adjustments and repeated measures. Nat. Methods 21, 83–91. doi: 10.1038/s41592-023-02092-7, PMID: 38158428 PMC10776411

[ref14] Liu Q. Mak J. W. Y. Su Q. Yeoh Y. K. Lui G. C. Ng S. S. S.. (2022). Gut microbiota dynamics in a prospective cohort of patients with post-acute COVID-19 syndrome. Gut 71, 544–552. doi: 10.1136/gutjnl-2021-325989, PMID: 35082169

[ref15] Lv A. Ma B. B. Z. Qiong D. Ma D. W. Z. Ma P. B. Z. Yao D.. (2024). Birth outcomes of pregnant women infected with COVID-19 in highland areas of China from 2020 to 2022: a retrospective analysis. Infect. Drug Resist. 17, 927–934. doi: 10.2147/IDR.S435751, PMID: 38481654 PMC10933517

[ref16] Nandi S. Ahmed S. Saxena A. Saxena A. K. (2023). “Exploring the pathoprofiles of SARS-COV-2 infected human gut–lungs microbiome crosstalks” in Probiotics, prebiotics, synbiotics, and postbiotics: human microbiome and human health. eds. Kothari V. Kumar P. Ray S. (Singapore: Springer), 217–235.

[ref17] Rizzello F. Viciani E. Gionchetti P. Filippone E. Imbesi V. Melotti L.. (2024). Signatures of disease outcome severity in the intestinal fungal and bacterial microbiome of COVID-19 patients. Front. Cell. Infect. Microbiol. 14:1352202. doi: 10.3389/fcimb.2024.1352202, PMID: 38510960 PMC10952111

[ref18] Robeson M. S. 2nd O'rourke D. R. Kaehler B. D. Ziemski M. Dillon M. R. Foster J. T.. (2021). RESCRIPt: reproducible sequence taxonomy reference database management. PLoS Comput. Biol. 17:e1009581. doi: 10.1371/journal.pcbi.1009581, PMID: 34748542 PMC8601625

[ref19] Tsounis E. P. Triantos C. Konstantakis C. Marangos M. Assimakopoulos S. F. (2023). Intestinal barrier dysfunction as a key driver of severe COVID-19. World J. Virol. 12, 68–90. doi: 10.5501/wjv.v12.i2.68, PMID: 37033148 PMC10075050

[ref20] Upadhyay V. Suryawanshi R. K. Tasoff P. Mccavitt-Malvido M. Kumar R. G. Murray V. W.. (2023). Mild SARS-CoV-2 infection results in long-lasting microbiota instability. mBio 14:e0088923. doi: 10.1128/mbio.00889-23 37294090 PMC10470529

[ref21] Veerus P. Nomm O. Innos K. Allvee K. Karro H. (2024). SARS-CoV-2 infection during pregnancy and perinatal outcomes in Estonia in 2020 and 2021: a register-based study. Acta Obstet. Gynecol. Scand. 103, 250–256. doi: 10.1111/aogs.14721, PMID: 37974467 PMC10823385

[ref22] Xie L. Luo G. Yang Z. Wu W. C. Chen J. Ren Y.. (2024). The clinical outcome of COVID-19 is strongly associated with microbiome dynamics in the upper respiratory tract. J. Infect. 88:106118. doi: 10.1016/j.jinf.2024.01.017, PMID: 38342382

[ref23] Yao Y. Cai X. Ye Y. Wang F. Chen F. Zheng C. (2021). The role of microbiota in infant health: from early life to adulthood. Front. Immunol. 12:708472. doi: 10.3389/fimmu.2021.708472, PMID: 34691021 PMC8529064

[ref24] Zaneveld J. R. Mcminds R. Vega Thurber R. (2017). Stress and stability: applying the Anna Karenina principle to animal microbiomes. Nat. Microbiol. 2:17121. doi: 10.1038/nmicrobiol.2017.121 28836573

[ref25] Zhang L. Xu Z. Mak J. W. Y. Chow K. M. Lui G. Li T. C. M.. (2022). Gut microbiota-derived synbiotic formula (SIM01) as a novel adjuvant therapy for COVID-19: an open-label pilot study. J. Gastroenterol. Hepatol. 37, 823–831. doi: 10.1111/jgh.15796, PMID: 35170078 PMC9115073

[ref26] Zhang D. Zhou Y. Ma Y. Chen P. Tang J. Yang B.. (2023). Gut microbiota dysbiosis correlates with long COVID-19 at one-year after discharge. J. Korean Med. Sci. 38:e120. doi: 10.3346/jkms.2023.38.e120, PMID: 37069814 PMC10111044

[ref27] Zuo T. Zhan H. Zhang F. Liu Q. Tso E. Y. K. Lui G. C. Y.. (2020). Alterations in fecal fungal microbiome of patients with COVID-19 during time of hospitalization until discharge. Gastroenterology 159, 1302–1310.e5. doi: 10.1053/j.gastro.2020.06.048, PMID: 32598884 PMC7318920

